# Physician versus non-physician delivery of alcohol screening, brief intervention and referral to treatment in adult primary care: the ADVISe cluster randomized controlled implementation trial

**DOI:** 10.1186/s13722-015-0047-0

**Published:** 2015-11-19

**Authors:** Jennifer R. Mertens, Felicia W. Chi, Constance M. Weisner, Derek D. Satre, Thekla B. Ross, Steve Allen, David Pating, Cynthia I. Campbell, Yun Wendy Lu, Stacy A. Sterling

**Affiliations:** Kaiser Permanente Northern California, 1800 Harrison, Oakland, CA 94612 USA; Division of Research, Kaiser Permanente Northern California, 2000 Broadway, Oakland, CA 94612 USA; Department of Psychiatry, University of California, San Francisco, 401 Parnassus Ave, San Francisco, CA 94143 USA; The Permanente Medical Group, Kaiser Northern California, 1201 Fillmore Street, San Francisco, CA 94115 USA

**Keywords:** Alcohol screening, Brief intervention for alcohol misuse, Primary care, Unhealthy alcohol use, Cluster randomized trial, Implementation

## Abstract

**Background:**

Unhealthy alcohol use is a major contributor to the global burden of disease and injury. The US Preventive Services Task Force has recommended alcohol screening and intervention in general medical settings since 2004. Yet less than one in six US adults report health care professionals discussing alcohol with them. Little is known about methods for increasing implementation; different staffing models may be related to implementation effectiveness. This implementation trial compared delivery of alcohol screening, brief intervention and referral to specialty treatment (SBIRT) by physicians versus non-physician providers receiving training, technical assistance, and feedback reports.

**Methods:**

The study was a cluster randomized implementation trial (ADVISe [Alcohol Drinking as a Vital Sign]). Within a private, integrated health care system, 54 adult primary care clinics were stratified by medical center and randomly assigned in blocked groups of three to SBIRT by physicians (PCP arm) versus non-physician providers and medical assistants (NPP and MA arm), versus usual care (Control arm). NIH-recommended screening questions were added to the electronic health record (EHR) to facilitate SBIRT. We examined screening and brief intervention and referral rates by arm. We also examined patient-, physician-, and system-level factors affecting screening rates and, among those who screened positive, rates of brief intervention and referral to treatment.

**Results:**

Screening rates were highest in the NPP and MA arm (51 %); followed by the PCP arm (9 %) and the Control arm (3.5 %). Screening increased over the 12 months after training in the NPP and MA arm but remained stable in the PCP arm. The PCP arm had higher brief intervention and referral rates (44 %) among patients screening positive than either the NPP and MA arm (3.4 %) or the Control arm (2.7 %). Higher ratio of MAs to physicians was related to higher screening rates in the NPP and MA arm and longer appointment times to screening and intervention rates in the PCP arm.

**Conclusion:**

Findings suggest that time frames longer than 12 months may be required for full SBIRT implementation. Screening by MAs with intervention and referral by physicians as needed can be a feasible model for increasing the implementation of this critical and under-utilized preventive health service within currently predominant primary care models.

Trial registration: Clinical Trials NCT01135654

## Background

Unhealthy alcohol use is a major contributor to the global burden of disease and injury [1, 2] and accounts for one in ten deaths of US adults aged 20–64 [3]. Screening and brief intervention to address non-dependent unhealthy alcohol use within primary care is effective [4–6] and recommended in national practice guidelines (including the U.S. Preventive Services Task Force (USPSTF) [7], the U.S. National Institute on Alcohol Abuse and Alcoholism’s (NIAAA) Clinician’s Guide “Helping Patients Who Drink Too Much” [8], and Canada’s College of Family Physicians Guideline [9]), but is seldom delivered in health care settings. Fewer than one in six Americans report being asked about or discussing their drinking with a health professional [10], and screening is rarely conducted in US primary care settings [11] outside the Veteran’s Affairs Health System (VA) [12]. Since efforts to implement alcohol Screening and Brief Intervention (for non-dependent unhealthy use, such as binge drinking with no current adverse consequences [13]) and Referral to Treatment (for alcohol dependence) (SBIRT) have fallen short [14], the field has called for more SBIRT implementation research [15–17]. This is particularly timely given important new environmental factors, such as the alcohol screening and brief counseling performance measures developed and approved by American Medical Association’s Physician Consortium for Performance Improvement, the Affordable Care Act’s provisions for substance use services, the Mental Health Parity and Addiction Equity Act, and Medicare reimbursement codes for SBIRT [18].

Studies of SBIRT implementation within primary care settings have found implementation difficult [19–21] and have typically focused on delivery of SBIRT by physicians. A European study of a tailored, multi-faceted improvement program for physician-delivered SBIRT [20] found little change in screening and intervention behavior at 12 months. Implementation of SBIRT delivery by primary care physicians, including brief advice, has been difficult to achieve even with financial incentives provided [19].

Non-physician provision of SBIRT may also be considered as a viable approach. Babor and colleagues found higher implementation rates by non-physicians and similar effectiveness to physician-delivered screening and brief intervention [22, 23]. Moreover, use of non-physician providers to conduct behavioral and disease management interventions is increasingly being considered as an appropriate and cost-effective alternative to physician delivery, and has been found to be acceptable to patients [24–29]. There is evidence that primary care teams involving nurses or health educators for behavioral interventions have higher rates of preventive screenings, such as colorectal cancer screening [30].

The Alcohol Drinking as a Vital Sign (ADVISe) study is a cluster, randomized trial in 54 adult primary care clinics within 11 Kaiser Permanente Northern California (KPNC) medical centers. It compares implementation of primary care physician (PCP)-delivered to non-physician provider-delivered SBIRT, and to usual care. In the non-physician provider-delivered arm, medical assistants (MAs) screen and clinical health educators, behavioral medicine specialists or registered nurses deliver brief intervention and referral to treatment (NPP and MA arm). The outcomes examined are screening rates and rates of brief intervention and referral to treatment (BI/RT) among those who screened positive. Cluster randomization of clinics prevented contamination between clinicians, and also was necessary because training and quality improvement is generally conducted at the clinic level. Because there is some variation in practice and leadership across the organization by medical center [31] and consistent with prior research on physician versus non-physician SBIRT implementation [22], we present findings overall and by medical center. Based on prior research showing that non-physicians can increase implementation of preventive and behavioral interventions, and findings that the most common barriers to SBIRT delivery in primary care are limited physician time and competing priorities [28, 32–35], we hypothesized that the NPP & MA arm would have higher rates of screening and BI/RT than the PCP arm.

To inform implementation strategies, we also examine how, within each intervention arm (PCP and NPP and MA), patient, physician, and system-level factors may be associated with SBIRT implementation as the literature suggests that such factors influence implementation [36, 37]. Prior research suggests that critical factors within these domains include provider training, time constraints [28, 32, 35], lack of treatment referral resources [38], systems issues such as staff workloads, patient demographic and clinical characteristics, and integration of screening into the electronic health record (EHR) [36, 39–41]. Thus, we examined how implementation outcomes were related to patient factors including demographic characteristics and medical and psychiatric problems [42], physician factors including training, demographics and specialty [28, 43, 44], and system factors [22, 45] including office visit length, the ratio of support staff to physicians, and co-location of specialty alcohol and drug (AOD) services [46].

Due to the naturalistic design of the current study we did not measure provider attitudes. However, recent research from a large European implementation trial [47, 48] suggests that providers’ role security with and commitment to addressing alcohol misuse are cross-sectionally related to reports of addressing alcohol misuse [47]. Yet, neither role security nor therapeutic commitment were prospectively related to screening or intervention [48].

To better understand the system context, we surveyed physician leaders within the study sites to understand alcohol screening practices and policies within the internal setting prior to the training and implementation processes. We hold constant a key factor found to be a salient influence on provider performance of care services—an EHR screening tool [49] made available in all three study arms. EHR use is growing steadily, both domestically [50, 51] and in many developed [52] and developing [53, 54] countries, increasing opportunities to incorporate such tools broadly. The findings on physician versus non-physician implementation and factors related to implementation within each delivery model can inform delivery and implementation of alcohol SBIRT and similar USPSTF-recommended behavioral care services [46] in primary care settings.

## Methods

### Setting

KPNC is a private, integrated health care delivery system covering 15 counties and serving 3.8 million members, [55] and KP covers 40 % of the insured population in California [56] with AOD services provided internally. The membership reflects the population of the region [57, 58], with more than 40 % of adult members being non-white or Hispanic, nearly 30 % with only a high school or lower level of education, and 44 % with annual incomes less than $65,000. KPNC’s and the University of California San Francisco’s institutional review boards approved the study.

### Randomization, sample and intervention

The unit of randomization was clinic. Eleven medical centers (of 47 in KPNC) were chosen to represent both urban and suburban locations and various clinic sizes. Fifty-four primary care clinics were in the 11 KPNC medical centers, located in Fairfield, Napa, Redwood City, San Francisco, San Jose, Santa Clara, Santa Rosa, South Sacramento, South San Francisco, Vacaville, and Vallejo. The 54 clinics were stratified by medical center and randomly assigned (parallel-group design) in blocked groups of three to SBIRT by physicians (PCP arm) versus non-physician providers and medical assistants (NPP and MA arm) versus usual care (Control arm), with 18 clinics in each of the three arms.

**Fig. 1:**
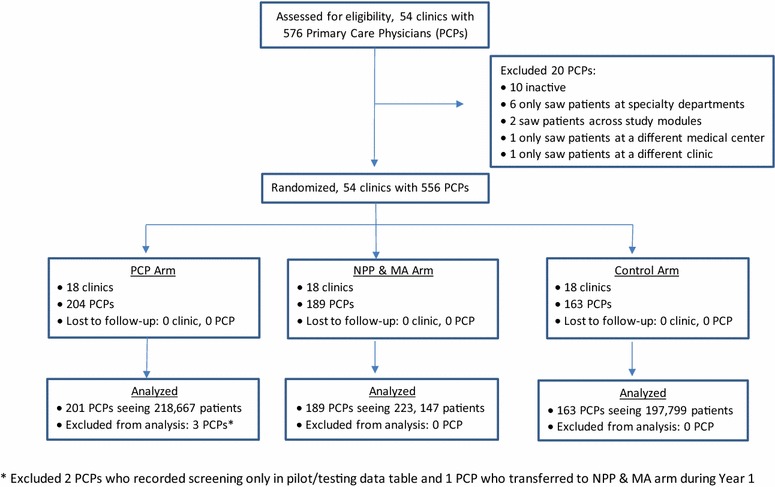
Flow diagram of participating clinics, physicians and patients through phases of the trial

There were 204 physicians in the PCP clinics, 189 in the NPP and MA clinics, and 163 in the Control arm clinics. We excluded two physicians in the PCP arm who used an earlier version of the screening tool (see methods below for screening tool details). In the year following the date at which each clinic had started the study (ranging from September 22, 2010 to November 24, 2010), a total of 218,667 unique patients had visits to the PCP arm physicians, 223,147 to the NPP and MA arm physicians, and 197,799 to the Control arm physicians (Fig. 1). Consistent with other studies [41, 59, 60], and because screening and interventions were intended to be part of usual care, we used KPNC’s EHR and other administrative data to examine patient and provider data and did not recruit individual providers or patients.

### Screening tools

The evidence-based [61] screening tool from the NIAAA Clinician’s Guide [8] was added to the adult primary care EHR with an automated clinical reminder at each adult (ages 18+) patient visit. It included the NIAAA single question screener “How many times in the past year have you had 5 or more drinks in a day” (for men aged 18–65, and “4 or more drinks” for women and individuals aged 66 and older) [8, 61]. Providers and MAs were instructed to use a laminated chart (distributed to exam rooms in the intervention arms) during screening to show patients various alcoholic beverage types and their standard drink sizes. Per the NIAAA Guide and prior studies, if a patient’s response was positive to the single question (i.e., >0 times exceeding the daily low-risk limit), the patient is considered an unhealthy drinker. Thus, for responses >0 times to this question, the tool prompted additional questions to assess use exceeding the NIAAA weekly low-risk limit (“On average, how many days a week do you have an alcoholic drink?” and “On a typical drinking day, how many drinks do you have?”), plus a validated two-question screener for alcohol use disorders (“In the past year, have you sometimes been under the influence of alcohol in situations where you could have caused an accident or gotten hurt?” and “Have there often been times when you had a lot more to drink than you intended to have?”; an affirmative response to either is considered a positive screening result) [62]. The EHR contained fields for recording whether a BI or RT was performed.

### Intervention

In both PCP and NPP and MA arms, providers were trained to deliver the same intervention, drawn from the NIAAA Guide. Patients who screened positive on the alcohol use disorders risk questions would be referred to AOD treatment for further assessment and possible treatment. Patients who screened positive on the unhealthy drinking questions but negative for alcohol use disorder risk would receive brief intervention consisting of providers stating their concern and advising them to cut back to low risk limits or abstain, as outlined in the NIAAA Guide [8]. Further, providers were trained in brief motivational intervention, and to: (1) incorporate salient medical conditions if possible (e.g., “drinking above these limits can worsen your hypertension and insomnia”), and (2) ask patients how ready they were to make the recommended changes, and (3) assist in goal-setting to reduce or stop drinking if the patient was willing. Patients were given the NIAAA publication “Tips for Cutting Down on Drinking” [63].

#### PCP arm

Physicians were trained to conduct all aspects of SBIRT.

#### NPP and MA arm

MAs were trained to ask the screening question, and non-physician providers were trained to ask the questions about weekly drinking and the two alcohol disorder screening questions and to conduct BI/RT. Chiefs of medicine and administrative managers at each site determined which type of non-physician provider would be trained to provide BI/RT. In Medical Centers 1, 6, 10, and 11, clinical health educators were trained; in Medical Centers 2, 3, 4, 5, and 9, behavioral medicine specialists (clinical psychologists or licensed clinical social workers) were trained; and in Medical Centers 7 and 8, registered nurses were trained. MAs roles include escorting patients to exam rooms, collecting and recording (in the EHR) health screening data, including blood pressure and smoking. They are required to have completed a vocational medical assisting training program and are supervised by nurse managers.

#### Control arm

Patients received usual care. Providers and MAs had access to the same EHR resources as in the intervention arms and physicians were invited to a 30-min webinar information session on the EHR evidence-based screening tool. Prior to the study, alcohol screening was not part of the organizational workflow. There was no defined screening or intervention policy or standardized workflow for either physicians or non-physicians to address unhealthy alcohol use in the Control arm. In the Control arm webinar information session, the screening tool was described as evidence-based but additional details (such as sensitivity and specificity) were not provided. The NIAAA daily and weekly low-risk limits were given. No information or training were given on BI/RT in the Control condition.

### Training

#### Training the trainers

Intervention training was adapted from the Alcohol Clinical Training (ACT) curriculum [64]. Trainers (internists, psychiatrists, psychologists, and social workers) attended one 4-h session on SBIRT and another 2-h session covering the KPNC-specific components including the EHR tool and referral information.

#### Training physicians and non-physician providers

PCP arm physicians and NPP and MA arm non-physician providers were given a 2-h training. The curriculum and duration of training were equivalent across arms; the trainings differed only in instruction about the workflow (with the NPP and MA arm including instruction about the handoff to the non-physician provider). In the NPP and MA arm, physicians were also included in the first hour of the training to learn about the importance and effectiveness of SBIRT and the workflow. The first hour included the NIAAA low-risk drinking limits [8], the USPSTF recommendations, the prevalence of unhealthy alcohol use in primary care in KPNC, evidence of the adverse health effects of unhealthy alcohol use, and effectiveness/cost-effectiveness of specialty treatment for alcohol, screening procedures and the screening tool (including sensitivity and specificity for identifying unhealthy alcohol use and a demonstration of the EHR screening tool); and an overview of the basics of the BI/RT and how to code for interventions/referrals. The second hour covered the BI/RT in more depth, including brief motivational interviewing, video case studies, and role-play practice. It also included a brief presentation by an AOD clinician about how to refer and assessment and treatment services offered at the local AOD clinic.

Each clinic also received in-person technical assistance 3 months after training. Trainers reviewed the clinics’ quality feedback reports on screening, intervention, and referral rates; and addressed questions and challenges. At 6 months, clinics received a 30-min “booster” training. All physicians in the PCP arm were included in the booster training. In the NPP and MA arm, physicians, NPPs and MAs were included.

#### MA training

In the NPP and MA arm, MAs were trained for one hour by registered nurses. The training included information about the health impact of unhealthy alcohol use, the effectiveness of SBIRT, the evidence-based screening questions and their ability to identify unhealthy alcohol use. MAs were provided with a demonstration of the EHR screening tool. They were given training on how to ask the screening questions in the EHR, which they practiced through role-play. The trainers also addressed “normalizing” alcohol screening and intervention (e.g., comparing with smoking and exercise) as integral to routine primary care services.

#### Training participation

In the PCP arm, 90 % of physicians attended training. In the NPP and MA arm, 100 % of non-physician providers, 85 % of MAs, and 80 % of physicians attended. For physicians who missed training, online, on-demand training was available. For MAs who missed training, their managers and senior peers provided training using the training materials. Brief videos on the EHR tool use were available to all on the EHR information and support intranet site. In the Control arm, a half-hour webinar session on use of the EHR screening tool was available on-demand via the KPNC intranet following the live session.

### Quality feedback reports

In the PCP and NPP and MA arms, quarterly reports were emailed to each clinic presenting their average screening and BI/RT rates and the names of the top five performing clinics within each arm with their mean rates.

### Leadership engagement

The CEO of The Permanente Medical Group (which employs the organization’s physicians) endorsed the project via a video introduction to the study training in which he discussed the links between unhealthy alcohol use and common health conditions and how SBIRT can benefit patients with unhealthy alcohol use. The Chair of the Chiefs of Adult Primary Care Medicine and the previous Chair (who retired a few months into the study) were both supportive and volunteered their clinics—“modeling” behavior for other Chiefs and physician colleagues. In the NPP and MA arm, clinic managers (to whom the MAs report) generally directed the MAs to use the screening tool, and it was generally seen as part of the MAs’ responsibilities. In each medical center, the physician chiefs of medicine (to whom the physicians report) agreed to having their sites used for the project and generally encouraged (but did not mandate) the use of SBIRT by physicians and non-physician providers.

### Incentives

Incentives to conduct SBIRT were limited to clinic recognition in the quality feedback reports (see “quality feedback reports” above) for high performing clinics. There were no financial incentives for any staff or providers to conduct screening, brief intervention, or referral to treatment.

### Data sources and measures

#### Primary care leader survey

We conducted an online survey of the Chiefs of Adult Primary Care in each medical center and the Physician Leader of each clinic, to understand practices and policies related to alcohol screening and intervention prior to the training. The response rate among the 62 physicians was 73 %. Participants were offered a $50 gift card. The survey also asked the allocated office visit length in the clinic which was used to calculate average visit time for each medical center.

#### EHR and administrative databases

The EHR was used to extract patient age, gender, and medical comorbidities. In the year prior to each patient’s index screening visit, we identified presence of a psychiatric diagnosis (i.e., psychoses, neurotic, personality and other psychiatric disorders, excluding AOD disorders) and presence of an other chronic disease diagnosis (yes/no) (i.e., Arthritis, Hypertension, Chronic Pain, Diabetes Mellitus, Asthma, Ischemic Heart Disease, Congestive Heart Failure, Stroke/Cerebrovascular Accident, Epilepsy, Parkinson’s Disease, End-Stage Renal Disease, HIV, Osteoporosis, Chronic Obstructive Pulmonary Disease (COPD) (ICD-9 codes available upon request). Physician race/ethnicity, gender, age, and MD specialty (Internal Medicine, Family Medicine or other) as well as the ratio of MAs to PCPs in each clinic were drawn from the administrative databases.

For each patient in the sample (i.e., who had one or more visits to a study physician in the study year), screening (1 if screened/else 0) was measured as having been asked the single-item screener as recorded in the EHR tool on any visit to the provider within the study year. If occurring in multiple visits within 1 year, the index screening visit was defined as the first visit. BI/RT was measured as having a visit in which an intervention or referral was recorded during or within 45 days of the screening visit for those who screened positive at the screening visit. We allowed a 45-day window because providers were trained to schedule a follow-up visit for the BI/RT within 6 weeks if unable to complete during the screening visit. We examined the EHR tool (containing fields for providers to record interventions and referrals) and also the diagnostic “V-code” for “Counseling, Alcohol Prevention” as indication of brief intervention or referral (as per the training). We also reported the BI and RT rates, separately, for each arm.

#### Other data collection

Co-location of primary care and specialty AOD clinics was measured by examining medical center maps and confirming with local staff. Anonymous questionnaires gathered at booster sessions asked providers and MAs the average amount of time spent on screening and asked providers the average total amount of time spent on BI/RT. To inform quantitative results, provider and staff feedback on the implementation and workflow challenges at the booster sessions was documented as part of data collection, and qualitative interviews with staff and providers at each study clinic were conducted.

### Analyses

Analyses were performed using SAS version 9.3 (SAS Institute Inc., Cary, NC, USA) and Stata version 10.1 (StataCorp, College Station, TX, USA). Physician survey results were examined descriptively. We calculated average monthly and annual screening rates for each medical center by study arm, and examined differences in screening rates between study arms using z statistics for comparisons of proportions. We also examined the number and percent screened for the best month over the 12 months after study start, which is the highest screening rate that each entity attained. Similarly, screening rates of BI/RT performed among those screened positive were calculated for each medical center and overall by study arm, and differences examined using Chi square tests. We also report a population-based BI/RT rate [65] by arm—this figure is the proportion of patients of all those with visits at the primary care clinics who received the intervention (regardless of being screened or not).

Within each intervention arm, multilevel logistic regression models were fitted to examine associations between patient-, physician- and system-level factors and screening and BI/RT implementation outcomes while accounting for clustering within physician and within clinic correlation of patient outcomes. Patient characteristics included age (18–24, 25–44, 45–59, and 60+), gender, and comorbid psychiatric and chronic conditions. Physician characteristics included age, gender, race/ethnicity, years serving the health plan, and specialty. System characteristics included co-location of primary care and AOD clinics, ratio of MAs to PCPs at the primary care clinic, average allocated office visit length, and number of physicians trained. For each outcome, we first fitted a series of multilevel univariate models to examine each predictor individually. All predictors associated with the outcome at p < 0.10 for either intervention arm were successively included in the multilevel models, with categories in the same direction being collapsed for some predictors to avoid extreme Odds Ratios (ORs) and 95 % Confidence Intervals (CIs) due to empty or small cells (for example, PCP race/ethnicity categories were collapsed to Non-White and White). Generalized Linear Latent and Mixed Models (GLLAMM) were used for multilevel analyses. We examined physician characteristics as predictors only in the PCP arm because SBIRT delivery in the NPP & MA arm was contingent on an entire team rather than an individual. No adjustments were made for multiple comparison [66].

## Results

### Screening practices prior to study implementation

In the survey of primary care leaders (N = 45) prior to the training, 88 % reported no policies or requirements to ask patients about alcohol. Those with policies indicated no consistent evidence-based screening methods.

### Screening rates and proportion screened positive

Table 1 shows the average number of unique visits, screens per month, average percent of patients screened and the number and percent screened for the best month over the 12 months after study start in each medical center and overall. For average percent screened, the NPP and MA arm (50.9 %) was higher (p < 0.0001) than both the PCP arm (9.2 %) and the Control arm (3.5 %); and the PCP arm higher (p < 0.0001) than Control. Exceptions were three medical centers (1, 3, and 11) in which rates were higher in the Control than the PCP arm. A similar pattern was found for the best screening rate. The NPP and MA arm had higher rates (60.1 %) than either the PCP (10.7 %) or Control arm (6.0 %), and the PCP arm had higher rates than the Control arm (p < 0.0001), with higher rates in the Control than PCP arm for Medical Centers 1, 3, and 11. Some movement of MAs from the NPP and MA arm to other arms occurred in these medical centers; in some sites MAs covered duties for absent MAs in other clinics.

**Table 1:** Screening rates by treatment arms in first year

	Average unique visits per month			Average screens per month			Average % screened			p value	Best month N screened			Best month % screened			p value
	PCP	NPP & MA	Control	PCP	NPP & MA	Control	PCP	NPP & MA	Control		PCP	NPP &MA	Control	PCP	NPP & MA	Control	
Medical center																	
1	1464	2797	2928	41	1107	148	2.80	39.56	5.07	^a,b,c^	161	1505	391	11.47	46.88	13.81	^a,b’,c^
2	1088	1424	1392	51	824	24	4.67	57.84	1.73	^a,b,c^	102	1039	68	9.31	66.65	5.00	^a, b,c^
3	925	748	1779	58	197	403	6.28	26.33	22.64	^a,b,c^	126	467	697	13.15	55.12	35.49	^a,b,c^
4	4318	4536	1639	552	2962	4	12.77	65.29	0.26	^a,b,c^	861	3681	23	19.79	71.71	1.18	^a,b,c^
5	1569	884	1717	509	411	107	32.43	46.51	6.22	^a,b,c^	635	723	235	37.90	64.21	14.61	^a,b,c^
6	3124	7668	3372	177	3705	29	5.68	48.32	0.85	^a,b,c^	332	5644	63	10.15	74.68	2.02	^a,b,c^
7	3336	2452	1898	339	225	6	10.17	9.16	0.33	^a,b,c^	980	636	25	26.04	29.42	1.49	^a,b,c’^
8	3866	4067	5686	565	2778	167	14.62	68.32	2.94	^a,b,c^	964	3355	350	28.41	75.48	5.81	^a,b,c^
9	5111	955	2531	405	142	8	7.92	14.86	0.33	^a,b,c^	608	254	41	12.36	31.59	1.76	^a,b,c^
10	4297	5034	6031	345	2827	79	8.03	56.17	1.32	^a,b,c^	637	3938	195	15.07	73.16	3.39	^a,b,c^
11	6636	3715	3125	239	2223	138	3.60	59.85	4.41	^a,b,c^	355	2586	443	5.04	70.78	15.08	^a,b,c^
All	35,519	34,167	31935	3280	17397	1113	9.23	50.92	3.49	^a,b,c^	3,729	20,445	1918	10.68	60.07	6.04	^a,b,c^

Differences in screening rates between each of the two intervention arms vs. Control arm as well as between the two intervention arms were examined by hand calculating z statistics for comparisons of two proportions

^a^p<0.0001, NPP & MA vs. Control; ^b^p<0.0001, PCP vs. Control; ^b’^p<0.05, PCP vs. Control; ^c^p<0.0001, NPP & MA vs. PCP; ^c’^p<0.01, NPP & MA vs. PCP

**Fig. 2:**
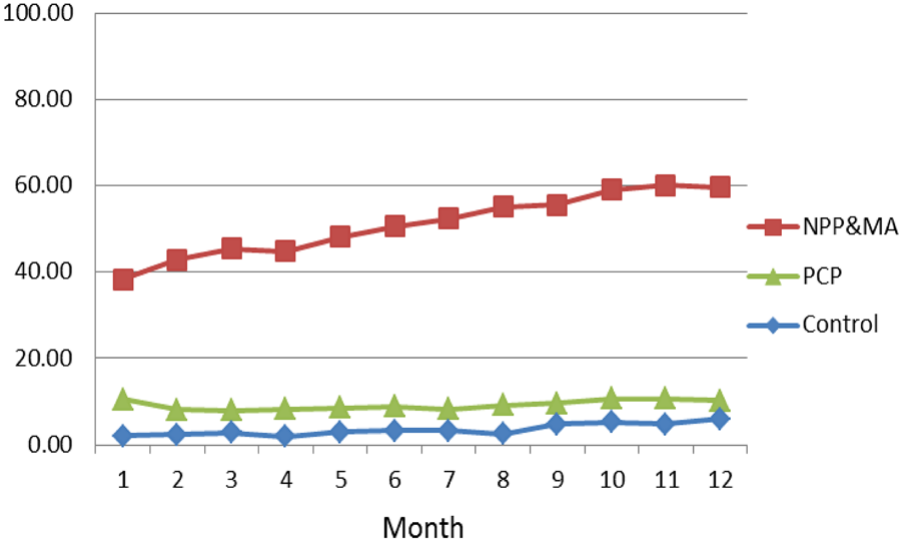
Average screening rates by month for NPP and MA arm, PCP arm and control arm

Figure 2 presents monthly average screening rates by arm across the 12 study months. NPP and MA arm rates increased from 38 % in month one to 60 % the final two months. PCP arm rates fluctuated between 8 and 10 % over time, and the Control arm had a small uptick from 2 to 6 %.

Of patients who were screened in the 12 months, the proportion screened positive for unhealthy drinking was 9.7 % in the PCP arm, 10.8 % in the NPP and MA arm, and 9.9 % in the Control arm.

### Intervention and referral rates among those screened positive for unhealthy alcohol use

Table 2 presents numbers and percentages of patients with positive screens who received BI/RT. The PCP arm had higher (p < 0.0001) BI/RT rates (44.4 %) than either the NPP & MA arm (3.4 %) or the Control arm (2.7 %); this pattern differed only for medical center 5 (with no difference between PCP and NPP and MA arms) and medical center 9 (NPP and MA arm had higher rates than PCP arm, p < 0.0001). Generally, BI/RT rates did not differ between NPP and MA and Control arms with the exceptions of medical center 5 with a slightly higher NPP and MA arm rate (p < 0.01) and medical center 8 with a slightly higher Control arm rate (p < 0.05). The percent of patients who received RT (not shown) was 9.8, 0.6 and 0.2 % of those screening positive in the PCP, NPP and MA and Control arms, respectively. We also examined the proportion of all patients with primary care visits (regardless of screening) who received the BI/RT. We found that 0.63, 0.24 and 0.02 % of patients seen in the PCP, NPP and MA, and Control arms, respectively, received the BI/RT (not shown).

**Table 2:** “Unhealthy drinkers” who received brief interventions or referral to treatment (BI/RT) by treatment arm

	PCP			NPP & MA			Control			p values^2^
	N screened positive	N received BI/RT	%	N screened positive	N received BI/RT	%	N screened positive	N received BI/RT	%	
Medical center										
1	90	51	56.67	1916	47	2.45	185	1	0.54	b, c
2	129	16	12.40	1849	24	1.30	41	1	2.44	b’’’, c
3	51	8	15.69	123	5	4.07	216	3	1.39	b’, c’’
4	241	141	58.51	1829	96	5.25	6	0	0.00	b’’, c
5	145	17	11.72	229	23	10.04	142	3	2.11	a’, b’’
6	246	120	48.78	2114	69	3.26	19	1	5.26	b’, c
7	394	200	50.76	172	9	5.23	26	0	0.00	b, c
8	503	153	30.42	1776	47	2.65	137	9	6.57	a’’, b, c
9	306	15	4.90	54	16	29.63	9	0	0.00	a’’’, c
10	697	545	78.19	4260	140	3.29	175	7	4.00	b, c
11	306	115	37.58	1259	55	4.37	176	5	2.84	b, c
All	3108	1381	44.43	15581	531	3.41	1132	30	2.65	b, c

^1^Chi-square tests were used to compare the proportions of patients screened between each of the two intervention arms vs. control as well as between the two intervention arms

^2^a: p<0.0001, NPP & MA vs. Control. a’ p<0.01, NPP & MA vs. Control. a’’: p<0.05, NPP & MA vs. Control. a’’’: p<0.10, NPP & MA vs. Control. b: p<0.0001, PCP vs. Control. b’: p<0.001, PCP vs. Control. b’’: p<0.01, PCP vs. Control. b’’’: p<0.10, PCP vs. Control. c: p<0.0001, NPP & MA vs. PCP. c’: p<0.01, NPP & MA vs. PCP. c’’: p<0.05, NPP & MA vs. PCP

Post-hoc analysis for medical center 9 found that a few physicians in the NPP and MA arm delivered BI/RT themselves to their few patients screening positive; these physicians were responsible for the vast majority of BI/RTs delivered in this clinic. Post-hoc analysis comparing subtype of non-physician provider found no clinically significant difference; rates of BI/RT for behavioral medicine specialists, clinical health educators, and nurses was 4.0, 3.3, and 2.9 %, respectively (not shown).

### Predictors of screening

Table 3 presents results of the multilevel models predicting screening in each of the intervention arms. Younger patients were less likely to be screened than patients over 60 years old in both arms. In both arms, women were less likely, and patients with psychiatric diagnoses and chronic medical conditions were more likely, to be screened.

**Table 3:** Patient-, physician- and system-level factors associated with delivery of screening by treatment arm

	PCP Arm					NPP & MA Arm				
	Coefficient value	Std. err.	OR	95% CI	p value	Coefficient value	Std. err.	OR	95% CI	p value
*Patient level*										
Age										
18–24 vs. ≥60	−0.1018	0.0430	*0.90*	*(0.83, 0.98)*	*0.018*	−0.1726	0.0656	*0.84*	*(0.74 , 0.96)*	*0.009*
25–44 vs. ≥60	−0.1582	0.0303	*0.85*	*(0.80, 0.91)*	*0.000*	−0.1329	0.0426	*0.88*	*(0.81, 0.95)*	*0.002*
45–59 vs. ≥60	−0.1074	0.0280	*0.90*	*(0.85, 0.95)*	*0.000*	−0.1319	0.0408	*0.88*	*(0.81, 0.95)*	*0.001*
*Gender*										
Female vs. Male	−0.2447	0.0217	*0.78*	*(0.75, 0.82)*	*0.000*	−0.2008	0.0305	*0.82*	*(0.77, 0.87)*	*0.000*
*Comorbid psychiatric conditions*										
Yes vs. no	0.1231	0.0269	*1.13*	*(1.07, 1.19)*	*0.000*	0.2436	0.0383	*1.28*	*(1.18, 1.38)*	*0.000*
*Chronic diseases*										
Yes vs. no	0.1934	0.0243	*1.21*	*(1.16, 1.27)*	*0.000*	0.3407	0.0345	*1.41*	*(1.31, 1.50)*	*0.000*
*Physician level*										
Age										
(per 1 year increase)	−0.2183	0.0034	*0.80*	*(0.80, 0.81)*	*0.000*					
Gender										
Female vs. male	1.1647	0.0247	*3.21*	*(3.05, 3.36)*	*0.000*					
Race/ethnicity										
Non-white vs. white	1.4355	0.0263	*4.20*	*(3.99, 4.42)*	*0.000*					
Years of services at the health plan										
(per 1 year increase)	0.2587	0.0032	*1.30*	*(1.29, 1.30)*	*0.000*					
Specialty										
Non-internal medicine vs. internal medicine	3.0877	0.0366	*21.93*	*(20.41, 23.56)*	*0.000*					
*System level*										
Colocation of AOD and primary care departments										
Yes vs. no	−2.3244	0.0689	*0.10*	*(0.09, 0.11)*	*0.000*	−0.4432	0.2274	*0.64*	*(0.41, 1.00)*	*0.051*
MA/PCP ratio at the clinic	−0.4393	0.0493	*0.64*	*(0.59, 0.71)*	*0.000*	3.0850	0.0748	*21.87*	*(18.89, 25.32)*	*0.000*
Ave. visit time	0.3606	0.0097	*1.43*	*(1.41, 1.46)*	*0.000*	0.1379	0.0104	*1.15*	*(1.12, 1.17)*	*0.000*
Number of PCPs trained at the clinic	−0.0387	0.0047	*0.96*	*(0.95, 0.97)*	*0.000*					

A three-level multivariate logistic model adjusting for clustering effects within physician and clinic was run for each of the two intervention arms

*PCP* primary care physicians, *NPP* non-physician providers, *MA* medical assistants, *AOD* alcohol and other drug

In the PCP arm, we examined physician-level factors associated with screening. Older physicians were less likely, and female physicians more likely, to screen than their counterparts. Non-White physicians had four times higher odds of screening than White physicians (OR = 4.20, 95 % CI 3.99, 4.42). Each year of employment in the health plan was associated with a 30 % increase in odds of screening (OR = 1.30, 95 % CI 1.29, 1.30). Physicians with Family Practice or other specialties were far more likely to screen than Internal Medicine physicians.

Regarding system characteristics, lower odds of screening were associated with co-located primary care and AOD clinics in the PCP arm. A higher ratio of MAs vs. physicians was associated with lower odds of screening for the PCP arm, but higher odds for the NPP and MA arm. Longer visit time was related to a higher likelihood of screening in both arms. In the PCP arm, having more physicians trained was related to a lower odds of screening. In the NPP and MA arm, all non-physician providers were trained, so we excluded this variable from the NPP and MA analysis.

### Predictors of brief intervention and referral among those screened positive for unhealthy alcohol use

Table 4 presents multilevel models of BI/RT among those screened positive in the intervention arms. Females were less likely to receive BI/RT in both. In the NPP and MA arm, patients with psychiatric or chronic conditions were more likely to receive BI/RT and those aged 45–59 years had higher odds than those aged 60 and older.

**Table 4:** Patient-, physician- and system-level factors associated with delivery of BI/RT by treatment arm

	PCP Arm					NPP & MA Arm				
	Coefficient value	Std. err.	OR	95% CI	p value	Coefficient value	Std. err.	OR	95% CI	p value
*Patient level*										
Age										
18–24 vs. ≥60	0.2127	0.2119	1.24	(0.82, 1.87)	0.315	0.3589	0.2019	1.43	(0.96, 2.13)	0.075
25–44 vs. ≥60	−0.1290	0.1755	0.88	(0.62, 1.24)	0.462	0.2557	0.1632	1.29	(0.94, 1.78)	0.117
45–59 vs. ≥60	−0.1571	0.1730	0.85	(0.61, 1.20)	0.364	0.5254	0.1615	*1.69*	*(1.23, 2.32)*	*0.001*
Gender										
Female vs. Male	−0.5081	0.1166	*0.60*	*(0.48, 0.76)*	*0.000*	−0.4701	0.1067	*0.62*	*(0.51, 0.77)*	*0.000*
Comorbid psychiatric conditions										
Yes vs. no	0.1489	0.1403	1.16	(0.88, 1.53)	0.289	0.2391	0.1121	*1.27*	*(1.02, 1.58)*	*0.033*
Chronic diseases										
Yes vs. no	−0.0359	0.1165	0.96	(0.77, 1.21)	0.758	0.2444	0.0995	*1.28*	*(1.05, 1.58)*	*0.014*
*Physician level*										
Age										
(per 1 year increase)	−0.0608	0.0161	*0.94*	*(0.91, 0.97)*	*0.000*					
Gender										
Female vs. male	0.2221	0.1932	1.25	(0.86, 1.82)	0.250					
Race/ethnicity										
Non-white vs. white	−0.5904	0.1599	*0.55*	*(0.41, 0.76)*	*0.000*					
Years of services at the health plan										
(per 1 year increase)	0.0614	0.0152	*1.06*	*(1.03, 1.10)*	*0.000*					
Specialty										
Non-internal medicine vs. internal medicine	−0.2045	0.1932	0.82	(0.56, 1.19)	0.290					
*System level*										
Colocation of AOD and primary care departments										
Yes vs. no	−0.1525	0.5258	0.86	(0.31, 2.41)	0.772	0.6106	0.4493	1.84	(0.76, 4.44)	0.174
MA/PCP ratio at the clinic	−3.1177	0.3265	*0.04*	*(0.02, 0.08)*	*0.000*	−0.4583	0.3325	0.63	(0.33, 1.21)	0.168
Ave. visit time	0.3111	0.0546	*1.36*	*(1.23, 1.52)*	*0.000*	0.0106	0.0529	1.01	(0.91, 1.12)	0.841
Number of PCPs trained at the clinic	−0.1695	0.0360	*0.84*	*(0.79, 0.91)*	*0.000*					

A three-level multivariate logistic model adjusting for clustering effects within physician and clinic was run for each of the two intervention arms

*BI/RT* brief intervention or referral to treatment, *PCP* primary care physicians, *NPP* non-physician providers, *MA* medical assistants, *AOD* alcohol and other drug

Older physicians were less likely, while those who had worked at the organization longer were more likely, to provide BI/RT. On the other hand, Non-White physicians were less likely to provide BI/RT than were White physicians. Gender and specialty did not predict BI/RT delivery.

In the PCP arm, a higher ratio of MAs vs. physicians and having more physicians trained on SBIRT was associated with lower odds of BI/RT. Longer visit time was related to higher BI/RT rates. No significant associations between rates of BI/RT and system-level factors were found in the NPP and MA arm.

## Discussion

### Pre-implementation SBIRT practices

Consistent with research suggesting a widespread lack of SBIRT implementation [10], systematic alcohol screening was not conducted as part of usual care in this organization prior to the study. For the few physician leaders who reported any policies, the recommended practice was to use questions embedded in the EHR system for which there is no evidence, and which are not included in guidelines [7]. These findings underline the need for formalized SBIRT implementation programs, incorporating the use of evidence-based instruments, to ensure that patients obtain optimal primary care treatment, including addressing alcohol as a potential complicating factor in many prevalent health conditions.

### Implementation by physicians versus non-physicians

In this cluster randomized controlled trial in adult PC, we compared implementation of alcohol SBIRT in three arms: physician-delivered, non-physician-delivered and Control. Consistent with prior research [22], screening rates were much higher in the NPP and MA arm than PCP arm, and both had higher rates than the Control arm. Generally, this pattern was true across all sites, with some exceptions, particularly one medical center in which MAs (who were trained as part of the NPP and MA arm) “floated” between the NPP and MA and Control arm clinics, resulting in similar screening rates. Two of the medical centers had slightly higher rates in the Control than PCP arm due to some MAs “floating” into the Control arm.

MAs are well-placed to provide alcohol screening and it is consistent with their role to collect and record vital signs in the EHR, including weight, height, blood pressure and (in KPNC) smoking screening and exercise [67]. The NPP and MA arm’s steadily improving screening rates from 38 % in month 1 to 60 % in month 12 suggests that barriers to screening that MAs may encounter are temporary and may be addressed through managers’ use of ongoing monitoring and performance feedback reports and practice. Improvements may also have been affected by booster sessions and technical assistance visits, which minimized discomfort with screening through reframing and normalizing it as a routine part of health care. Normalizing was accomplished by acknowledging that drinking is common in our culture and not problematic if within low-risk limits, and by comparing alcohol screening to asking about smoking and exercise—both are routinely addressed in KPNC’s primary care clinics.

Different approaches to screening may also be more efficient and increase rates in the NPP and MA delivery model. The two NPP and MA clinics with the highest rates attributed success to the use of paper forms provided to patients by the receptionist and collected by MAs, who simply recorded responses in the EHR screening form; an approach they already used for other screening activities. This “paper and pencil” screening may increase workflow efficiency and increase quality and reliability of screening [68]. It may be that PCPs were less likely to consistently record screening when it was performed because of their many competing priorities, whereas MAs were more likely to record screening in the EHR tool because they were more closely supervised and monitored.

The BI/RT results were counter to prior research which found good implementation rates among non-physicians [22]. The PCP arm rates were 44.4 % versus the NPP and MA arm’s 3.4 %. This was the pattern in most medical centers, with one exception where two physicians provided interventions themselves. When examining a population-based intervention rate (i.e., the proportion of all patients seen who received BI/RT), the PCP arm still had an advantage (0.63 versus 0.24 %).

The lower BI/RT rate in the NPP and MA arm was counter to our hypothesis that non-physician providers would have higher BI/RT rates. Several factors may have contributed to low BI/RT rates in the NPP and MA arm. First, because this was a naturalistic implementation study, we trained non-physician providers working in the health system rather than using research or externally or grant-funded clinicians, who may have different characteristics and motivations (because of being paid by a research study and invested in the success of the intervention) rather than having an existing operational role with competing duties. Non-physician providers were not always available immediately and thus the workflow often required an additional appointment either by phone or in person, or required phoning the provider when possible via a “consult phone”, which meant a delay. Second, exam room availability was often an issue, which translated into concerns about backlogs if interventions were provided there. Third, providers reported that patients were resistant to seeing the non-physician providers, regardless of whether they were told that the discussion would be about their alcohol screening results or whether a more vague script about “healthy lifestyle” was used.

Overall, the findings from the current study suggest an efficient, but less complex workflow of MA screening followed by BI/RT by physicians, consistent with how other preventive services are delivered in this health system [69]. It also parallels recent findings that alcohol screening by staff and follow-up by clinicians is associated with more frequent use of screening and intervention [70]. These findings are also consistent with aspects of the Consolidated Framework on Implementation Research (CFIR) model [45]. The CFIR suggests that evidence-based interventions with greater complexity (such as the NPP and MA delivery model examined here) are less likely to be implemented. Also consistent with the CFIR framework, the lack of resources in the form of sufficient available time of non-physician providers and exam rooms were barriers to implementation of interventions and referrals in the NPP and MA arm. Further, the CFIR suggests that high compatibility of new evidence-based interventions with existing roles and duties can increase implementation as is the case for adding alcohol screening for MAs who already ask about other health behaviors (smoking and exercise).

One potential disadvantage with the physician-delivered intervention is that physicians reported shorter interventions (5 min average) versus non-physicians (23 min average). Yet longer interventions may not significantly increase effectiveness [71]. Physicians reported that the single item screening question required less than 1 min. We did not gather more specific information on how long providers spent on brief interventions versus referrals to specialty treatment.

These results may raise questions about the ability of currently predominant primary care models to use non-physician clinical team members to perform SBIRT and other new evidence-based practices. Team-based care has potential for addressing behavioral health problems including unhealthy alcohol and tobacco use and depression and for improving health conditions affected by these problems such as hypertension and obesity [72, 73]. Yet, a recent study documented staffing models in primary care practices which participated in a nationwide US team-based care initiative and found that even among those designated as patient-centered medical homes, fewer than 23 % employed health educators, pharmacists, social workers, nutritionists or community service coordinators and fewer than half employed care coordinators [74]. Ideally, medical home models and integrated care systems would include adequate levels of behavioral health care staff, on-call and readily available to deliver behavioral health interventions such as multiple-session brief interventions for unhealthy alcohol use [75]. Further, sufficient exam room space is needed to prevent backlogs, and the purpose of exam rooms may need to be expanded so that care coordination and behavioral services can be provided. Alternatively, primary care office design may need to be reconsidered so that sufficient non-physician provider consult room spaces are interspersed next to exam rooms.

In addition to increased team staffing, performance measures linked to incentives could aid implementation. The CFIR model and the experience at the US VA Health System, where alcohol SBIRT is mandated by an internal performance measure [41, 76], suggest that policies such as performance measures may increase implementation. Yet to date there are no external performance measures applicable to private, capitated systems or to US Federally Qualified Health Centers. Recently released findings that fewer than one in six Americans reported being asked about or discussing their alcohol consumption with a health professional are striking, given that the USPSTF has recommended it in general medical settings since 2004 [7]. In the system studied here, MA screening and physician intervention were found to be a feasible solution for implementation of SBIRT. Without structural changes in primary care practices that would improve their ability to provide consistent team-based care, MA to physician workflow may be one viable approach, and may also be considered for other underutilized USPSTF recommended services [46] such as depression screening. Full implementation of SBIRT likely also requires a time frame of more than 1 year for both individual providers and organizations to progress through stages of readiness for change in order to achieve optimal results.

### Predictors of implementation within each model

In both PCP and NPP and MA arms, younger patients were less likely to be screened; this was counter-intuitive given their higher prevalence of unhealthy drinking [77, 78] and also counter to prior research [79]. Similarly, in general, younger patients were not more likely to receive BI/RT, though in the NPP & MA arm, patients aged 45–59 who screened positive were more likely to receive BI/RT than those aged 60 or older. Physicians may assume that unhealthy drinking is “to be expected” in younger patients, and thus not be as concerned about identifying it. Also, the training emphasized the link between alcohol and chronic conditions, which may have made it easier for physicians to screen and discuss drinking among older patients at risk for such conditions.

Consistent with results suggesting that men are more likely to binge drink, male patients were more likely to be screened and receive BI/RT in both arms. This is consistent with prior research indicating that men are more likely to be screened [79–81] and raises concerns given that women are less likely to seek alcohol intervention and treatment and may be more vulnerable to adverse health effects of unhealthy use [82]. That more individuals with psychiatric and chronic disease diagnoses were screened than those without (in both arms) is also consistent with prior research [79] and with the emphasis in the provider training on the relationship of unhealthy alcohol use to adverse health effects and reduced medication adherence [83]. However, psychiatric and chronic conditions only predicted receiving BI/RT in the NPP and MA arm. Such patients are generally more likely to see non-physician providers for chronic conditions, e.g., for behavioral interventions related to diabetes, so the intervention may have been paired with conversations about their chronic condition.

As found in prior research, female physicians were more likely than male to screen [44, 84], though there was no difference by physician gender in providing BI/RT. Physicians from non-White and Latino ethnic/racial groups were more likely to screen, although White physicians were more likely to record providing BI/RT. Few studies have examined these characteristics as predictors of SBIRT; the large sample size and racial/ethnic diversity of the provider sample allowed us to examine this. More research is needed to understand these differences in screening and BI/RT rates between gender and racial/ethnic groups. For example, some national studies have found higher rates of alcohol abuse in whites than some other ethnic groups and in men than women [85, 86]. Perhaps these differences in screening may reflect that physicians with healthier habits themselves (which was unmeasured in this study) are more likely to provide counseling [87]. Alternatively, the findings may reflect gender and ethnic differences in communication styles in which case additional or different approaches to training may particularly be needed for these subgroups.

Consistent with a greater focus on preventive care, a specialty of Family Medicine or training other than Internal Medicine was related to screening, but this characteristic did not affect BI/RT rates. Physicians newer to the system were less likely to both screen and intervene. Perhaps longer experience in the system may help physicians to practice more efficiently, leaving time for preventive screening and intervention.

Consistent with studies suggesting lack of screening is due to time constraints [28, 88], longer visits were related to higher odds of screening in both arms and intervention in the PCP arm. Provision of BI/RT was not related to appointment time in the NPP and MA arm likely because the intervention usually required a separate visit. However, having a higher ratio of MAs per physician increased the likelihood of screening in the NPP and MA arm. Having more MAs did not seem to off-load the work of physicians enough in the PCP arm to result in higher rates of screening or intervention as we had expected, and in fact was related to a lower odds of screening and BI/RT. This may be because a higher MA ratio is a reflection of a busier clinic.

Some findings on system-level predictors were counter-intuitive. Having more physicians trained in SBIRT in a medical center was related to lower odds of screening. The findings that co-location between primary care and specialty AOD treatment clinics was associated with a lower likelihood of receiving screening or intervention are also counter to the ideas that proximity of specialty treatment would increase awareness and that easier patient access to specialty treatment would increase physician confidence in discussing alcohol with patients. More research is needed to understand how co-location affects primary care clinician behavior, and whether there is a confounding factor related to both variables.

### Study strengths and limitations

Conducting this study in real-world busy clinics, and using existing staff are important characteristics of an implementation study, and at the same time enhance both limitations and strengths. We could not control the schedules or priorities of clinic managers or providers or the movement of staff across clinics. However, using existing staff is important for understanding whether a model is feasible and sustainable regardless of externally funded interventionists.

Provider factors not examined include outcome expectancies, concerns about intrusiveness, beliefs that SBIRT is ineffective, negative attitudes about patients with alcohol problems, and self-efficacy; these have been previously well-documented [65, 79, 89–97].

Similar to studies for other health conditions, use of EHR data means that our data on screening and BI/RT are limited to that documented in the EHR. Yet this approach allowed for studying the natural process of care, studying a large number of patients, and having a population-based sample of patients and providers, rather than limiting our sample to those willing to be recruited into a study.

This study is the first to examine implementation of the NIAAA Guide for SBIRT [8] in adult primary care in a private, non-profit US health care system. It also uses an established EHR, a mechanism recommended by the field [15, 46], and known to be salient in predicting better implementation of primary care practices [98–101]. EHR use is also more generalizable to the future of private US health care settings because of Phase III Meaningful Use requirements [102].

Because of the large sample size, some statistically significant differences may not be clinically meaningful. Variations in the pattern of results across medical centers highlight the importance of including multiple medical centers or clinics when studying factors that influence implementation.

A further limitation is that data on alcohol use disorder screening were not recorded for a considerable proportion of those who screened positive for unhealthy alcohol use. We thus were unable to examine the proportion of those screening positive on the alcohol use disorders screen who received RT.

Finally, it is not known how the study’s findings would generalize to systems with fewer exam room constraints or systems in which clinical health educators are dedicated to SBIRT (which would allow for same-time, warm hand-offs to exam room consultations). Moreover, currently predominant primary care models do not include health educators or behavioral specialists [74].

## Conclusions

Our findings from this randomized trial of physician versus non-physician alcohol SBIRT implementation suggest that a model of MA screening and physician intervention may have the highest odds of implementation in currently predominant primary care models. Further, in large organizations such as this one, where policies and practices for systematic screening were not in place prior to training, implementation may take a longer time period than 1 year after an initial training and a higher “dosage” of quality improvement and feedback; longer time frames may be required for provider change [17] and full implementation, as has been found in the VA [68]. Many facilitators of implementation, such as organizational and provider stages of readiness for change [45] may increase over time after a new initiative begins. Informed by this study, a model using MA screening and physician intervention was adopted by Kaiser Permanente Northern California across all adult primary care clinics in June of 2013 for annual, evidence-based alcohol screening and intervention or referral to specialty treatment [103]. Future research is needed across systems that have implemented SBIRT to examine duration of time since initial implementation and duration and amount of related quality improvement activities as factors predicting SBIRT implementation outcomes. Research is also needed on whether implementation of BI and RT by non-physicians is more effectively implemented within fully realized and staffed medical home models.

## Abbreviations

AOD: alcohol and other drug
BI: brief intervention
EHR: electronic health record
MA: medical assistant
NIAAA: US National Institute on Alcohol Abuse and Alcoholism
NPP: non-physician provider
PCP: primary care physician
RT: referral to treatment
SBIRT: screening, brief intervention and referral to treatment
USPSTF: United States Preventive Services Task Force
